# Midline Anterior Neck Inclusion Cyst in a Pediatric Patient: A Case Report and Literature Review with a Dermatologic Perspective

**DOI:** 10.3390/medicina61010064

**Published:** 2025-01-02

**Authors:** Noemi Brigenti, Rachele Bardelli, Giovanni Paolino, Elisabetta Danese, Paolo Gisondi, Nicola Zerbinati, Giampiero Girolomoni, Andrea Carugno

**Affiliations:** 1 Section of Dermatology and Venereology, Department of Medicine, University of Verona, 37126 Verona, Italy; noemi.brigenti@studenti.univr.it (N.B.); rachele.bardelli@asst-settelaghi.it (R.B.); elisabetta.danese_02@studenti.univr.it (E.D.); paolo.gisondi@univr.it (P.G.); giampiero.girolomoni@univr.it (G.G.); 2 Dermatology Unit, Ospedale di Circolo Fondazione Macchi, ASST Sette Laghi, 21100 Varese, Italy; nicola.zerbinati@uninsubria.it; 3 Unit of Dermatology, IRCCS Ospedale San Raffaele, 20132 Milan, Italy; paolino.giovanni@hsr.it; 4 Department of Medicine and Innovation Technology (DiMIT), University of Insubria, 21100 Varese, Italy; 5 Department of Medicine and Surgery, University of Insubria, 21100 Varese, Italy

**Keywords:** Midline Anterior Neck Inclusion Cyst (MANIC), pediatric, developmental anomalies, congenital anomaly

## Abstract

Midline Anterior Neck Inclusion Cysts (MANICs) are rare congenital anomalies caused by improper embryonic fusion. These superficial benign lesions typically appear yellowish and cystic without deeper anatomic connections. We describe an 11-month-old boy with a stable, asymptomatic, yellow, elastic cystic lesion on the midline of the anterior neck, measuring 4 mm and present since shortly after birth. Clinical, dermoscopic, and ultrasound evaluations confirmed the diagnosis of MANIC. Over six months of observation, the lesion remained stable without growth, infection, or symptoms. MANICs are benign epidermoid cysts with minimal risk of complications that are often mistaken for thyroglossal duct cysts, dermoid cysts, or other congenital anomalies. Unlike thyroglossal duct cysts, they do not move during swallowing or tongue protrusion. Management is usually conservative, with surgery reserved for symptomatic or cosmetically significant cases. This case highlights the importance of parental reassurance and avoiding unnecessary intervention for asymptomatic lesions. Recognition of MANICs is essential for dermatologists and pediatricians evaluating midline neck lesions. A conservative approach with regular monitoring ensures optimal care while minimizing interventions. Further research may clarify the pathogenesis and long-term outcomes of these rare lesions.

## 1. Case Report

An 11-month-old boy was referred to the dermatology clinic with an asymptomatic, yellowish, elastic, and cystic lesion located on the midline of the anterior neck. The child, born via natural childbirth after an uncomplicated pregnancy, had no comorbidities and had met all expected psychomotor milestones. According to his parents, the lesion had been present since shortly after birth, remaining stable in size, color, and texture over time without causing discomfort, pain, inflammation, or drainage.

**Figure 1:**
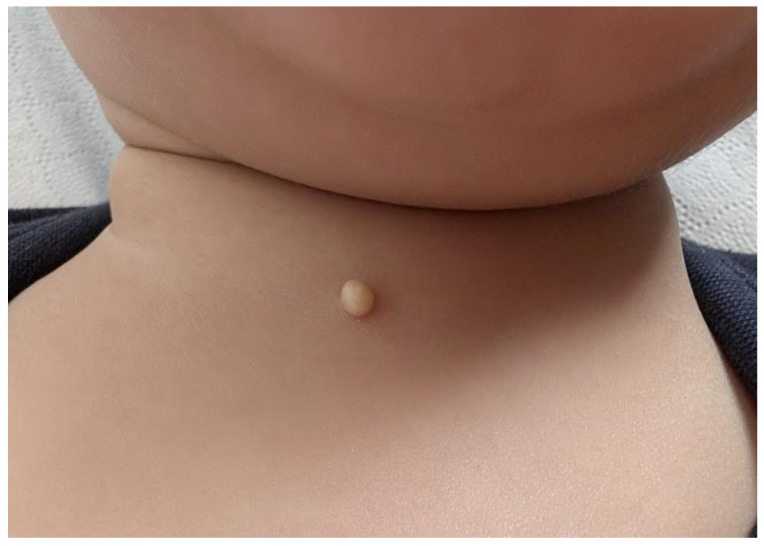
An 11-month-old baby with a rounded, yellowish cyst measuring 4 mm in the suprasternal region.

Physical examination revealed a smooth, rounded, yellowish, pedunculated bump, 4 mm in size, located in the suprasternal region (Figure 1). The lesion was mobile and elastic to palpation. Dermoscopy revealed a homogeneous white-yellowish papule without vascular pattern, pigmentation, or irregular features suggestive of malignancy or inflammation. Ultrasonography revealed a finely encapsulated cystic lesion of skin origin in the central anterior neck with no underlying fistulae, sinus tracts, or blood flow. Ultrasonography revealed a finely encapsulated cystic lesion of skin origin in the central anterior neck with no underlying fistulae, sinus tracts, or blood flow.

The diagnosis of the Midline Anterior Neck Inclusion Cyst (MANIC) was proposed based on the lack of movement during swallowing, the median suprasternal location, the absence of surrounding skin abnormalities, and the exclusion of deeper structural involvement. Informed of the management options, the parents chose a conservative approach, opting for regular follow-up instead of surgery for their newborn. Over a six-month period, the lesion remained stable with no evidence of growth, infection, or new symptoms.

MANICs are considered benign congenital anomalies and are generally managed conservatively unless they cause symptoms or significant cosmetic concerns. Spontaneous resolution or minimal progression is often observed [1]. In this case, the decision to follow up rather than surgically intervene was consistent with established recommendations for asymptomatic congenital cysts [1]. This case highlights the importance of recognizing and appropriately managing midline neck lesions, including MANICs, in pediatric patients. Dermatologists may be consulted for such conditions, and it is critical to emphasize the value of conservative management and parental reassurance when the clinical presentation supports a benign diagnosis.

## 2. Materials and Methods

A literature review was conducted from inception to November 2024 in PubMed and Embase databases. The following keywords were searched on PubMed and Embase: [‘MANIC’] OR [‘midline anterior neck inclusion cyst’]. Articles not pertinent to the topic were excluded. All identified reports were carefully evaluated for their relevance from a dermatological perspective. The reference sections of the selected articles were also reviewed to identify additional pertinent reports. Data from the included studies were analyzed to investigate the clinical and pathological characteristics of MANIC cases reported in the literature.

In addition to the literature review, we propose a practical guide for dermatologists to help differentiate between MANIC and other congenital neck anomalies with similar cutaneous features. This guide, which can be found in the following sections, aims to assist clinicians in distinguishing between the main differential diagnoses of congenital neck lesions that present with features resembling those of MANIC.

## 3. Discussion

Improper fusion during embryologic development of the anterior neck can result in several congenital midline neck anomalies [1]. Although rare, these conditions are significant developmental anomalies and account for a significant proportion of pediatric cervical masses [1].

This article examines MANICs from a dermatologic perspective, highlighting the role of the dermatologist in the initial clinical evaluation of these lesions. Although definitive management may be outside the scope of dermatologic practice, early recognition by the dermatologist is critical.

Congenital midline neck masses can result from a variety of conditions, including branchial malformations, thyroglossal duct cysts, dermoid or epidermoid cysts, cervical thymic cysts, cervical clefts, juvenile xanthogranulomas, and teratomas [2].

For the sake of completeness, it is also important to mention other congenital lesions that may occur in the central part of the neck in the pediatric population, such as vascular and lymphatic anomalies, malignant neoplasms, and infectious or inflammatory masses [2]. Key aspects of the clinical presentation, symptoms, and complications of these conditions are summarized in Table 1.

### 3.1. Branchial Malformations

The branchial apparatus is comprised of arches, grooves and pouches [3]. In the event that these structures fail to resolve and fuse, communication may be established between the skin and mucosa, resulting in the formation of a fistula. Alternatively, a sinus tract may develop, or in some cases, no communication may occur between the skin or mucosa, resulting in the formation of a cyst [4,5]. Although generally benign, these malformations can present unique clinical challenges due to the potential for superinfection, local compressive effects, and surgical complications [3]. The origin of this structure can be traced back to the branchial apparatus, a complex structure critical to the development of the head and neck that appears during early embryogenesis [3,4]. Abnormalities in this apparatus can lead to a spectrum of congenital malformations, which may manifest differently depending on the branchial arch involved. These malformations can range from asymptomatic cysts to symptomatic sinuses and fistulas with a propensity for infection and drainage [4].

The ectoderm is the most external of the three embryonic layers and develops into many of the outer layers of the body, including the epidermis, hair, nails, oral epithelium, cornea and olfactory epithelium, while the mesoderm is the intermediate layer of development between the ectoderm and endoderm, giving rise to the skeleton, muscle, heart and bones [3]. By the fourth stage of embryonic development, six paired branchial arches have formed in the human embryo. Each arch consists of a mesenchymal core covered externally by ectoderm and internally by endoderm and eventually differentiates into structures essential for head and neck anatomy [2]. Based on their location, these branchial cleft anomalies are classified into first, second, third and fourth types, each with distinct anatomical features and clinical presentations [3].

Of all branchial anomalies, the first branchial anomalies are relatively uncommon and are divided into two distinct types, originating in the ectoderm and mesoderm, respectively [6,7]. Type I anomalies originate from ectodermal tissue and represent duplications of the external auditory canal (EAC). Type II anomalies are more prevalent than Type I anomalies, as they involve both ectodermal and mesodermal components. Such anomalies may originate from the EAC, the middle ear cleft, or the nasopharynx. They are often characterized by a fistula that extends from the concha, the EAC, or the neck, running medially and inferiorly to the EAC [6,7].

Second branchial anomalies represent the most prevalent type. Anatomical location is the basis for classification of these anomalies into four categories. Type I anomalies are situated along the anterior edge of the sternocleidomastoid muscle, while Type II anomalies are in proximity to major blood vessels. Type III anomalies traverse the region between the internal and external carotid arteries, while Type IV anomalies, which are rare, are positioned medial to the great vessels [8]. Third branchial anomalies are encountered less frequently and may be difficult to differentiate from fourth branchial anomalies. Both such anomalies may manifest as neck abscesses or acute thyroiditis, with a higher incidence on the left side. Furthermore, these anomalies may also pose a risk of upper airway obstruction in neonates. It is worth noting that fourth branchial anomalies, although extremely rare, typically present during childhood [9,10].

### 3.2. Thyroglossal Duct Cysts

Thyroglossal duct cysts (TDCs) represent the most common congenital anomaly of the neck; this anomaly occurs in approximately 7% of people, representing about 75% of the congenital masses of the neck [11]. Although they are most diagnosed in the first decade of life, TDCs can also present in adults [11]. These cysts originate from a remnant of the thyroglossal duct, an epithelial tract that persists during the thyroid’s migration from the foramen cecum to its final anterior neck position. The persistence of this duct can result in the formation of cysts, sinuses, or fistulae. Thyroglossal duct cysts manifest in a variety of anatomical locations, with the majority (65%) situated in an infrahyoid, paramedian position. The four most common locations are the thyrohyoid (60.9%), suprahyoid (24.1%), supra-sternal (12.9%), and intra-lingual (2.1%) regions [12,13]. In clinical practice, TDCs typically present as non-tender, mobile masses. Infection of the cyst may manifest in a number of symptoms, including the presence of tenderness, dysphagia, dysphonia, fever, or a progressively enlarging neck mass, often triggered by an upper respiratory tract infection [14].

### 3.3. Dermoid or Epidermoid Cysts

Dermal cysts develop from both ectodermal and mesodermal components and therefore include sebaceous glands, hair follicles and sweat glands. A small percentage of dermal cysts occur in the head and neck region; most dermal neck cysts occur in the supraioid and suprasternal areas. In contrast to dermal cysts, epidermal cysts arise from ectodermal tissue alone and are found superficially in the subcutaneous tissue [15]. These cysts usually present as slow-growing midline masses that gradually increase in diameter over the years due to the accumulation of cutaneous products [15]. The standard treatment is simple excision, though midline dermoid cysts can extend deeper and may require more complex surgical approaches [16].

### 3.4. Thymic Cyst

Thymic cysts have their origin in the persistence of the embryologic thymopharyngeal duct, which is due to incomplete regression of the thymic primordium during the early stages of fetal development, specifically around the eighth week [15]. These cysts, composed of ectopic thymic tissue, are typically found in the anterior or deep regions of the sternocleidomastoid muscle. However, they can also appear along the jawline or extend to the thoracic inlet and mediastinum. Most cases are located on the left side of the neck and are more common in males. Generally, thymic cysts are asymptomatic and present as painless, slow growing, unilocular masses [17]. Should they become significantly enlarged, they may lead to compressive symptoms such as stridor, breathing difficulties, or dysphagia. Surgical excision is the primary treatment, with an excellent prognosis and no reported recurrences following complete removal [18].

### 3.5. Cervical Cleft

The midline cervical cleft is an uncommon congenital anomaly of the anterior neck with no significant gender preference [19]. Its clinical presentation is characterized by an erythematous, vertical, atrophic defect along the neck’s midline, devoid of adnexal structures [5]. Key features include a subcutaneous fibrous band that often extends beyond the visible skin defect, a skin tag at the superior margin, and a blind sinus inferiorly. The primary treatment modality for this condition is surgical correction, which has the dual objective of improving both functional and aesthetic outcomes [20,21].

### 3.6. Teratomas

Cervical teratomas are a rare type of germ cell tumor that originate from the germ cells of the head and neck. They predominantly occur in the cervical region and can affect newborns, causing respiratory distress due to airway obstruction [15]. Clinically, teratomas can present as well-defined masses with cystic and solid areas, containing structures such as cartilage and bone. Surgical excision is the primary treatment option, although it is a complex procedure due to the potential risks of tissue infiltration and recurrence [22].

### 3.7. Juvenile Xanthogranuloma

Juvenile xanthogranuloma (JXG) is a rare, benign form of non-Langerhans cell histiocytosis characterized by well-defined papules or nodules that appear erythematous or yellow orange, with a smooth surface and firm consistency, typically located on the head and neck. Most lesions manifest during the first year of life, and approximately one-third may be congenital. Congenital forms often present as large, solitary tumors with atypical morphology, including infiltrative plaques, deep subcutaneous nodules, or exophytic tumors. They present as solitary lesions in 80% of cases [23]. Common complications include ulceration and atrophic scarring, more frequently observed in larger lesions. Cutaneous lesions are self-limiting, typically resolving spontaneously, while extracutaneous involvement, although rare, most commonly affects the eyes [23].

Dermoscopically, JXG exhibits a distinctive “setting sun” pattern, characterized by a central yellow-orange area surrounded by an erythematous halo. Other dermoscopic findings may include peripheral linear telangiectasias, pale yellow deposits resembling “clouds”, and occasionally a faint pigment network or whitish streaks. These features can aid in the non-invasive diagnosis of JXG and differentiate it from other lesions [23].

### 3.8. Midline Anterior Inclusion Neck Cyst (MANIC)

MANICs are rare congenital anomalies of the anterior neck, recently described as a new entity in a case series by Walsh et al. [1]. These lesions are thought to result from improper embryonic fusion during development, leading to the formation of epidermoid inclusion cysts in the midline [3]. Congenital head and neck malformations are the result of a multifactorial process involving a complex interplay of genetic and environmental factors. Although the exact mechanisms underlying the development of MANICs remains unclear, general insights into the pathogenesis of neck malformations suggest that genetic predispositions, such as specific gene variations associated with craniofacial development, may play a pivotal role [24]. Environmental influences, including maternal smoking, alcohol consumption, and the use of certain medications during pregnancy, are known to exacerbate the risk [24].

They are typically superficial and mobile, with no deeper connections to underlying structures. When performed, the ultrasound usually shows a thin-walled cystic lesion arising from the dermal layer [1,25]. The ultrasound may help the clinician to rule out associated internal anomalies, invasion into deeper subcutaneous structures and increased surrounding vascularity. These lesions typically present histologically as dermal cysts lined by stratified squamous epithelium demonstrating maturation through a well-formed granular layer, filled with abundant orthokeratotic keratin and scattered hair shafts [1,25,26,27]. Walsh et al. [1] presented the initial case series of seven patients with these lesions, which resembled giant milium cysts and were not associated with deeper congenital defects. Subsequently, other authors have presented single case reports of similar lesions, thereby corroborating the proposed epidemiological, clinical and prognostic characteristics described in the aforementioned case series, which also manifest in our case [3,25,26,27,28,29].

As detailed in aforementioned articles and in our patient, the lesions were present at birth, and there was no family history of similar lesions. From the data, there appears to be no gender prevalence (five males, six females and two unknowns among the 13 cases considered). In all cases, the lesions presented as yellow-white or white papules with a diameter of approximately 2–7 mm. The location was consistent across all cases, situated on the lower midline of the anterior neck, just above the sternal notch. The clinical appearance was consistent with that of a giant milia. The mean age of presentation of the cases described in the cases reported in this article was 7.3 months (standard deviation 4.1 months), with an age range of 11 weeks to 15 months. Table 2 describes and summarizes all these characteristics.

## 4. Differential Diagnosis

In terms of its clinical appearance, its location and the age of onset, MANICs can be considered alongside thyroglossal duct cysts, cervical midline clefts, teratoma, branchial cleft cysts and bronchogenic cysts in the differential diagnosis [1]. Thyroglossal duct cysts, the most common congenital neck mass, arise from remnants of the thyroglossal duct and are typically located along the midline, often moving with tongue protrusion. This movement is a distinguishing feature from branchial cleft anomalies, which usually do not exhibit this characteristic [13,20]. Thyroglossal duct cysts are typically subcutaneous and occur along any point of the midline, but rarely at the suprasternal notch [16]. Midline cervical clefts present with a distinct overlying skin abnormality, characterized by erythema and a nipple-like projection at the superior aspect [19,21]. Branchial cleft cysts usually present as a non-tender, fluctuant mass along the anterior border of the sternocleidomastoid muscle [21]. Thyroglossal duct cysts and branchial cysts lack overlying skin change and appear as subcutaneous masses. The surface of the branchial sinuses and preauricular sinuses exhibit distinctive alterations in the dermal structure, accompanied by underlying anatomical variations. Dermoid and epidermoid cysts are similar to thyroglossal duct cysts in terms of quality and location. They are typically well-circumscribed, non-tender, and midline. However, in contrast to TDCs, they do not typically elevate with tongue protrusion or swallowing [13,21]. MANICs differ from JXG for clinical characteristics. A MANIC typically presents as a benign, fluctuant, asymptomatic white-yellow cyst located along the anterior midline of the neck, often near the hyoid bone, unlike JXG, which manifests as a solid nodule or papule with a distinctive yellow-orange coloration in addition to the presence of dermoscopic features described above [23]. Moreover, histologically, MANIC has features compatible with an epidermoid cyst without histiocytic proliferation or the characteristic Touton giant cells seen in JXG [23].

## 5. Management and Conclusions

From the cases in the literature, the management of MANICs is typically conservative, with surgery only employed in cases of symptoms or significant cosmetic concerns. In cases where the patient is asymptomatic, as in the present case, clinical observation with regular follow-up is the preferred approach, as the literature reports a low incidence of complications or significant progression in these benign lesions [1]. While an ultrasound is not a routine requirement for small lesions, it may be employed to confirm the nature and superficial location of the cyst when the diagnosis remains unclear. When performed, the ultrasound typically reveals a well-defined hypoechoic cyst situated within the superficial skin layers, consistent with the epidermoid nature of these lesions [1,25,26,27]. In a pediatric patient, it is essential to identify diagnoses that may require targeted and immediate intervention, as well as the need for additional diagnostic testing, prompt treatment, or active surveillance and follow-up [30]. In the future, advances in ultrasound technology (e.g., high-frequency ultrasonography), may further aid in the characterization of benign lesions, thereby facilitating non-invasive monitoring and improving follow-up strategies [31,32,33]. In the coming years, in addition to advances in the sensitivity of imaging technologies, artificial intelligence tools may prove invaluable in achieving increasingly precise diagnosis of neck neoplasms and malformations [34,35].

The literature suggests that MANICs are typically benign and stable with low risk of complications [1,25,26,27,28,29]. However, potential long-term sequelae include secondary infection, scarring, excessive growth, and persistent aesthetic concerns that may affect self-esteem during adolescence due to the central location of the lesion [1]. Most reported cases, including ours, involve pediatric patients under the age of one year [1,25,26,27,28,29]. Therefore, longer follow-up is crucial to fully understand long-term outcomes and to ensure timely intervention for complications or unexpected developments. Parental education and reassurance are equally important, while further research is needed to clarify the natural history of the condition and refine management strategies. As mentioned, surgical treatment may be considered in cases where the cyst becomes symptomatic, develops recurrent infections, or is perceived as a cosmetic concern by patients and their families. The surgical outcomes are typically favorable, with a minimal risk of recurrence or complications [1]. In our patient, in agreement with the parents, we opted for clinical follow-up and observed the stability of the lesion over a six-month follow-up period without significant changes. This approach was supported by the absence of symptoms or signs of growth, which highlighted the importance of parental reassurance and the necessity to avoid unnecessary interventions. Each case should be evaluated individually, with the consideration of specific circumstances and the presence of risk factors.

In the context of pediatric congenital anomalies, it is imperative to consider the potential psychological implications that a congenital disease, such as MANICs, can have on parents. Congenital anomalies, particularly if it is located close to vital structures such as the cervical neurovascular bundle or the thyroid gland, can cause considerable anxiety in parents [36]. A multidisciplinary approach is also recommended, involving different specialists such as otolaryngologists and pediatric surgeons to provide comprehensive care. A clear diagnostic framework and evidence-based management strategies are essential not only to guide clinical decisions, but also to reassure families, reduce stress, improve the overall quality of care and reduce feelings of disorientation among families [36].

In conclusion, MANICs are benign congenital lesions that rarely indicate the necessity for surgical intervention. In the majority of cases where there are no symptoms, a conservative approach involving regular monitoring is appropriate. This favors a minimal intervention strategy and provides parental support. Although dermatologists are not typically involved in the treatment of these lesions, their ability to recognize and promptly assess MANICs is essential. An early and accurate diagnosis, conducted in collaboration with pediatricians, can significantly impact the diagnostic pathway and patient management. Furthermore, an understanding of these lesions enables dermatologists to determine the optimal timing for eventual intervention and ensuring appropriate patient care. Further studies may enhance our understanding of the pathogenesis and long-term outcomes of these rare lesions.

## Figures and Tables

**Table 1:** Summary of Congenital Cervical Masses: Clinical Presentation and Management. This table provides an overview of various congenital cervical masses, focusing on their frequency, dermatological clinical presentation, and general treatment approach. The table highlights the most common features dermatologists may encounter and specific traits to aid in distinguishing between similar presentations.

Type of Congenital Cervical Mass	Clinical Presentation		Symptoms	Possible Complications
Branchial Anomalies	First arch anomalies	Periauricular swelling, sinus formation in the neck or external auditory canal, ear discharge, or a fistula below the mandible angle	All these malformations can range from asymptomatic cysts to symptomatic sinuses and fistulas	Infection, drainage and otologic complications
	Second arch anomalies	Mass or fistula along the sternocleidomastoid muscle or be located near major vessels		May acutely increase in size during an upper respiratory tract infection, leading occasionally to respiratory compromise
	Third arch anomalies	Recurrent low-neck abscesses, acute suppurative thyroiditis, and neck masses		Risk of upper airway obstruction in neonates and infections
	Fourth arch anomalies			
Thyroglossal Duct Cyst	Mobile midline mass that moves with swallowing or tongue protrusion due to connection with the hyoid bone		Usually painless but can become tender if infected	Possible infections
Dermoid/Epidermoid Cysts	Well-circumscribed, mobile, midline mass; lacks movement with tongue protrusion. No overlying skin changes		Usually asymptomatic	Possible infection and/or drainage
Thymic Cyst	Slow-growing mass, typically on the left side of the neck; extends toward the thoracic inlet or mediastinum		Usually painless and asymptomatic mass	Stridor or dysphagia if large
Cervical Cleft	Erythematous, vertical, atrophic defect along the neck’s midline, subcutaneous fibrous band, a skin tag at the superior margin and a blind sinus inferiorly		The area frequently exhibits serous discharge	The blind sinus tract may discharge mucoid material; and the subcutaneous cordlike fibrous may cause webbing
Teratoma	Well-defined mass with cystic and solid areas that may contain cartilage and bone. Its size and symptoms may distinguish it from other benign masses.		Often causes respiratory distress due to airway obstruction	Feeding difficulties, neck deformities, and vascular or nerve compression
Juvenile xanthogranuloma	Well-defined red and yellow or orange papule with smooth surface; solitary lesion in 80% of cases.		Usually asymptomatic	Atrophic scarring and ulceration, more common in large exophytic lesions
Midline Anterior Neck Inclusion Cyst (MANIC)	Small, superficial, mobile cyst with no deep attachments, usually on lower anterior neck		Non-tender, with stable appearance over time, aiding differentiation from inflammatory lesions or infected cysts	Severe respiratory distress and dysphagia due to tracheal and esophageal compression

**Table 2:** Summary of reported cases of congenital Midline Anterior Neck Inclusion Cyst (MANIC). The table summarizes the main clinical features of 12 cases described in the literature and our newly reported case. For each case, the following information is provided: patient sex, family history, age of lesion appearance (present at birth in all cases), age at clinical presentation, clinical course, associated symptoms and age at removal or biopsy (if applicable). Most cases exhibited a stable course or limited lesion growth over time, with symptoms generally absent or mild. One case showed spontaneous resolution with residual scarring.

Source of Case	Sex	Family History	Age of Appearance	Age at Presentation	Course	Associated Symptoms	Age at Removal or Biopsy
Walsh R et al. [1]	M	None	Birth	2 months	Slight growth over time	None	13 months
Walsh R et al. [1]	M	None	Birth	15 months	Growth	Some pulling and scratching	15 months
Walsh R et al. [1]	M	None	Birth	11 weeks	No changes	None	16 months
Walsh R et al. [1]	F	None	Birth	6 months	No changes	None	6 months
Walsh R et al. [1]	M	None	Birth	7 months	No changes	None	n/a
Walsh R et al. [1]	F	None	Birth	4 months	Commensurate growth	None	n/a
Walsh R et al. [1]	F	None	Birth	2 months	Spontaneous resolution with residual scar	None	n/a
Rushin C et al. [25]	F	None	Birth	10 months	No changes	None	10 months
Frigerio A et al. [26]	F	None	Birth	11 months	Growth	Lesion was mildly irritating	11 months
Miller E et al. [27]	n/a	None	Birth	6 months	Growth	None	n/a
Wong XL et al. [28]	F	None	Birth	11 months	n/a	None	n/a
Alfaro-Sepúlveda D et al. [29]	n/a	n/a	n/a	n/a	n/a	n/a	n/a
Our case	M	None	Birth	11 months	No changes	None	n/a
